# Supplementary material for: KCNK18 Biallelic Variants Associated with Intellectual Disability and Neurodevelopmental Disorders Alter TRESK Channel Activity
Source: Int J Mol Sci. 2021 Jun 4;22(11):6064. doi: 10.3390/ijms22116064 (PMC8200030; doi:10.3390/ijms22116064)
Supplement: Supplementary file 1 [file ijms-22-06064-s001.zip › ijms-1214922-supplementary.pdf]

Supplementary materials

KCNK18 Biallelic Variants Associated with Intellectual Disability and Neurodevelopmental Disorders Alter TRESK Channel Activity

Lisa Pavinato[1,2], Ehsan Nematian-Ardestani[3], Andrea Zonta[1], Silvia De Rubeis[4,5,6,7], Joseph Buxbaum[4,5,6,7,8,9], Cecilia Mancini[10], Alessandro Bruselles[11], Marco Tartaglia[10], Mauro Pessia[3,12], Stephen J. Tucker[13], Maria Cristina D'Adamo[3*#], Alfredo Brusco[1*#]

Supplementary Table S1. Prediction of variants impact using in silico tools

	p.(Tyr163Asp)	*p.(Ser252Leu)*
GnomAD allele frequency	0	0.0001379 (39/282862)
CADD GRCh37-v1.6	23.9	22.9
DANN	0.9909	0.9991
BayesDel addAF	Damaging	Tolerated
BayesDel noAF	Damaging	Damaging
EIGEN PC	Pathogenic	Pathogenic
DEOGEN2	Damaging	Tolerated
FATHMM-MKL	Damaging	Damaging
FATHMM-XF	Damaging	Neutral
LRT	Deleterious	Deleterious
MutationTaster	Disease causing	Disease Causing
PROVEAN	Damaging	Damaging
SIFT	Damaging	Damaging
SIFT4G	Damaging	Damaging
